# The impact of social media use intensity on college students’ self-esteem: the mediating roles of online group identity and online selective exposure

**DOI:** 10.3389/fpsyg.2026.1729680

**Published:** 2026-05-29

**Authors:** Yifei Li, Minmin Chen, Qiang Zhang, Juan Li, Jie Zhang, Jingping Zhang, Yuxuan Zhang

**Affiliations:** 1 Xiangya School of Nursing, Central South University, Changsha, Hunan, China; 2 Xiangya Stomatological Hospital & Xiangya School of Stomatology, Central South University, Hunan, China; 3 School of Mechanical and Electrical Engineering, Henan University of Technology, Zhengzhou, China; 4 Hunan University of Chinese Medicine, Changsha, Hunan, China

**Keywords:** college students, online group identity, online selective exposure, self-esteem, social media use intensity

## Abstract

**Objective:**

This study explores how the intensity of social media use influences self-esteem among college students, focusing on the mediating roles of online selective exposure and online group identity. It draws on Social Identity Theory and Selective Exposure Theory to better understand these relationships.

**Methods:**

A cross-sectional survey was conducted with 4,789 college students, selected through cluster random sampling. Two representative universities were chosen from each of China’s Central, Eastern, and Western regions. Participants completed validated questionnaires measuring social media use intensity, online selective exposure, online group identity, and self-esteem. Restricted cubic spline (RCS) analysis was employed to explore potential nonlinear relationships between social media use intensity and self-esteem, based on the hypothesis that the relationship may not be linear. Chain mediation analysis with 5,000 bootstrap samples was performed to evaluate the indirect effects of online selective exposure and online group identity.

**Results:**

The findings revealed a positive linear relationship between social media use intensity and self-esteem. Both online selective exposure and online group identity played partial mediating roles in this relationship. Specifically, higher social media engagement was associated with stronger identification with online groups and greater exposure to content consistent with one’s self-concept, which in turn were linked to higher self-esteem.

**Conclusion:**

The study suggests that social media engagement can positively influence college students’ self-esteem through enhanced online group identification and selective exposure to self-relevant content. These results provide empirical evidence for the mechanisms underlying the association between social media use and self-evaluative outcomes among young adults.

## 1 Introduction

Social media has become an integral component of college students’ daily lives. According to recent data from Kepios, approximately 5.24 billion people worldwide use social media, representing about 63.9% of the global population (Kemp, 2025). Moreover, 79% of individuals aged 15 to 24 are internet users (Union, International Telecommunication, 2025), indicating the extensive penetration of social media among younger cohorts. In China, the number of social media users has reached 1.107 billion, accounting for 78.6% of the total population (China Internet Network Information Center, 2025). Evidence from other surveys of Chinese college students further shows that more than half spend over 3 h per day on social media platforms (Zhang and Liu, 2025). For this population, social media serves not only as a means of maintaining social connections and accessing information but also as a key environment in which self-concept is formed. This pervasive engagement links social media use to students’ psychological development: while it provides opportunities for social interaction and emotional support, it may also influence self-evaluation through algorithmic information exposure and feedback mechanisms (Bayer et al., 2018; Gonzales and Hancock, 2011; Valkenburg and Peter, 2007). As self-esteem represents a central aspect of personality and psychological adjustment (Pyszczynski et al., 2004), understanding how social media use affects self-esteem is essential for addressing the mental health needs of contemporary college students.

The relationship between social media use and self-esteem has attracted extensive scholarly attention, however, existing findings remain notably inconsistent (Cingel et al., 2022). This divergence largely stems from differing theoretical perspectives on the psychological mechanisms through which social media influences self-esteem. Social comparison theory (Festinger, 1954) posits that individuals evaluate their self-worth through comparisons with others, which provides a central framework for understanding the potential negative effects of social media on self-esteem. Within social media contexts, idealized self-presentations and highly visible social information readily trigger upward social comparisons. These comparisons may lead to self-devaluation or feelings of social exclusion and ultimately undermine self-esteem (Carraturo et al., 2023; Ozimek and Bierhoff, 2020; Schmuck et al., 2019). In contrast, self-affirmation theory (Steele, 1988) emphasizes individuals’ motivation to maintain self-integrity and suggests that social media offers important contextual resources for self-expression, value affirmation, and the acquisition of positive feedback. When individuals encounter social or psychological threats, positive interactions and affirming feedback can help restore self-integrity. This process may enhance self-esteem and subjective well-being (Khoo et al., 2024; Toma and Hancock, 2013). Against the backdrop of these theoretical divergences, some studies further propose that the relationship between social media use and self-esteem may be nonlinear, such as an inverted U-shaped pattern (Cingel and Olsen, 2018). Specifically, moderate levels of social media use may facilitate self-affirmation, whereas excessive use may amplify negative effects due to algorithm-driven exposure to comparison-oriented information (Cingel and Olsen, 2018). Nevertheless, although prior research has examined the association between social media use and self-esteem from multiple theoretical perspectives, a systematic and integrative conceptual pathway explaining how social media affects self-esteem through specific psychological mechanisms remains insufficiently developed.

In response to the aforementioned theoretical divergences, social identity theory and selective exposure theory provide valuable perspectives for further clarifying the psychological pathways through which social media influences self-esteem. Social identity theory (Zhang and Zuo, 2006) posits that self-esteem is partly derived from individuals’ identification with their social groups, emphasizing that individuals maintain positive self-evaluations by internalizing group membership as an integral component of the self-concept. This perspective offers an important theoretical foundation for understanding the mediating role of online group identification in the relationship between social media use and self-esteem. Meanwhile, selective exposure theory (Festinger, 1954; Knobloch-Westerwick, 2014) suggests that individuals tend to seek out information that is consistent with their existing beliefs, attitudes, and self-concepts in order to reduce cognitive dissonance and maintain self-consistency. This process may influence self-evaluations through the cumulative effects of informational valence. Building on these perspectives, the present study integrates social identity theory and selective exposure theory to propose a serial mediation mechanism involving online group identification and online selective exposure, which together explain the pathway linking social media use to self-esteem.

Specifically, at moderate levels of engagement, frequent social media use facilitates individuals’ contact with and integration into online groups, while the formation and strengthening of group identification provide a fundamental value orientation and evaluative framework for information selection (Ellison et al., 2007). As group identification increases, individuals become more inclined to engage with information that aligns with group norms and values and, through sustained positive interaction feedback, reinforce their sense of self-worth, thereby enhancing self-esteem (Cingel et al., 2022; Gonzales and Hancock, 2011). During this process, the presence of information cocoons may further intensify these psychological mechanisms by reinforcing repeated exposure to group-congruent content (Pariser, 2011). However, when social media use intensifies beyond a certain level, excessive online engagement may weaken individuals’ connections with offline social contexts (Primack et al., 2017). Under such conditions, even if psychological benefits derived from online group identification and selective exposure persist, they may be insufficient to offset the potential negative consequences associated with reduced participation in real-world social interactions, thus constraining or diminishing the positive impact of social media use on self-esteem (Andreassen et al., 2017; Primack et al., 2017).

In summary, this study proposes a progressive psychological transmission pathway linking social media use to self-esteem, namely, social media use intensity → online group identification → online selective exposure → self-esteem. Online group identification refers to individuals’ sense of belonging, emotional attachment, and psychological identification with specific online communities or groups, whereas online selective exposure refers to individuals’ tendency to seek out information that is consistent with their beliefs, attitudes, and self-concepts while avoiding conflicting content (Zhang and Zhao, 2022). Although prior studies have separately examined the mediating roles of online group identification or selective exposure (Aubrey, 2006; Barker, 2009), the serial mediation mechanism formed by these two processes has not yet been systematically tested. This study is the first to examine their serial mediation in the context of social media use and self-esteem, offering new insights into how these processes interact to shape self-esteem.

Based on the above theoretical framework, the following hypotheses are proposed:

*H1*: Social media use intensity has a nonlinear relationship with college students’ self-esteem.

*H2*: Online group identification and online selective exposure are both positively associated with college students’ self-esteem.

*H3*: Online group identification and online selective exposure mediate the relationship between social media use intensity and college students’ self-esteem.

## 2 Method

### 2.1 Design

This study adopted a cross-sectional survey design and was conducted in accordance with the STROBE guidelines for observational studies (Wiehn et al., 2021). A cluster random sampling strategy was used. Based on information from the Chinese Ministry of Education and official university websites, a list of comprehensive universities was identified. Comprehensive universities were defined as institutions offering a full range of academic disciplines and providing both undergraduate and postgraduate education. Considering the three major geographic regions of China (Eastern, Central, and Western), 2 universities were randomly selected from each region as sampling clusters. Restricting the sample to comprehensive universities ensured institutional comparability, while including universities from different regions improved geographic representation. Data were then recruited and collected from these universities between September and November 2024. The electronic questionnaire was administered via the Wenjuanxing online platform. After providing informed consent, participants voluntarily completed and submitted the survey.

### 2.2 Participants

Inclusion criteria were: (1) current enrollment as a college student and (2) possession of a mobile device capable of completing the online questionnaire. All participants provided written informed consent after being fully briefed on the study’s purpose and procedures. Exclusion criteria included: (1) no current neurological or psychiatric disease (Kazdin, 2015; Rouault et al., 2022), (2) those who had previously participated in similar studies or were concurrently engaged in other related research, to avoid carry-over effects arising from prior experimental interventions or survey experiences.

### 2.3 Sample size

The sample size was determined based on three core principles: (1) structural equation modeling (SEM) typically requires a minimum of 200 participants (Wu, 2010); (2) the sample size should be at least 20 times the number of observed variables (Wu, 2010). In this study, 10 observed variables were included, comprising 7 demographic variables and three factors: Online Selective Exposure, Online Group Identity, and Social Media Use Intensity. This calculation method indicated a minimum sample size of 200 participants. (3) According to Harrell’s recommendation, models with 3–7 knots yield optimal fitting performance, and the required sample size is calculated to exceed 240 using the formula *N* ≥ 40 × (*k* − 1) (Harrell, 2015).

After accounting for an estimated 20% invalid response rate, the minimum required sample size was determined to be 288. A total of 5,210 questionnaires were distributed. After excluding 421 invalid responses due to reasons such as failure to meet the inclusion criteria, excessively short response times (less than 200 s), or patterned response behaviors (including failure on verification items such as “Please select ‘A’ for this question”), 4,789 valid responses remained for subsequent data analysis. This resulted in an effective response rate of 91.9%. The final valid sample of 4,789 participants far exceeded the minimum required threshold, thereby ensuring that the statistical power requirements for all planned analyses were met.

### 2.4 Data collection

An online questionnaire was used for data collection in this study, administered through the Wen Juanxing platform from September to November 2024. Given that this study involved multiple regions, an online survey format was chosen to facilitate efficient data collection across different locations. To ensure reliability, the survey instrument underwent a pilot test with 30 college students in August 2024, where the scales were evaluated for applicability, and a minimum response time of 200 s was established. For the formal survey, a list of comprehensive universities was first identified based on information from the Chinese Ministry of Education and official university websites. Considering the three major geographical regions of China (Eastern, Central, and Western), two universities were randomly selected from each region as sampling clusters. Emails containing the study information and questionnaire link were distributed to students through university contacts, and participants were encouraged to share the link with peers. The questionnaire link was distributed via email and snowball sampling methods.

Additionally, participants who were unwilling to sign the electronic informed consent form or self-reported having a mental illness were instructed to skip the questionnaire entirely. This exclusion criterion was implemented to ensure the validity and reliability of the data, as individuals with mental health conditions may have different cognitive and emotional responses that could potentially distort their self-reports on the survey (Kazdin, 2015). After data collection, the questionnaires were evaluated, and those completed in less than 200 s were excluded from the analysis due to concerns about the carefulness of completion. All questionnaires were completed anonymously, and the data are securely stored on equipment in our laboratory to prevent any potential data leakage.

### 2.5 Measurements

#### 2.5.1 General information questionnaire

The research team developed the general information questionnaire following an extensive review of the existing literature. The questionnaire covered sociodemographic variables, including gender, age, grade, major, only-child, place of residence, and left-behind experience during childhood.

#### 2.5.2 Social media use intensity scale

The social media use intensity scale developed by Zhang and Zhao (2022) was employed to assess the intensity of participants’ social media use. The scale comprises five items (e.g., “Social media platforms such as Weibo, WeChat, and TikTok have become a part of my daily life”) and uses a 5-point Likert scale ranging from 1 (strongly disagree) to 5 (strongly agree). Higher scores reflect a greater intensity of social media engagement. The original scale demonstrated good internal consistency, with a Cronbach’s alpha of 0.881. In this study, the Cronbach’s alpha was 0.915.

#### 2.5.3 Online Selective Exposure Scale

The Online Selective Exposure Scale developed by Zhang and Zhao (2022) was used to assess participants’ tendency to engage with information that aligns with their existing beliefs and attitudes. The scale consists of five items and employs a 5-point Likert scale ranging from 1 (strongly disagree) to 5 (strongly agree). Higher scores indicate a stronger tendency toward selective exposure. The original scale demonstrated good internal consistency, with a Cronbach’s alpha of 0.917. In the present study, the Cronbach’s alpha was 0.915, indicating satisfactory reliability.

#### 2.5.4 Online Group Identity Scale

The Online Group Identity Scale was employed to assess participants’ sense of identification with online communities and the extent to which they perceive social validation from like-minded peers (Zhang and Zhao, 2022). The scale consists of five items and employs a 5-point Likert scale ranging from 1 (strongly disagree) to 5 (strongly agree). Higher scores reflect a stronger sense of online group identification. The original scale demonstrated good internal consistency, with a Cronbach’s alpha of 0.849. In the present study, the Cronbach’s alpha was 0.912, indicating satisfactory reliability.

#### 2.5.5 Rosenberg Self-Esteem Scale (RSES)

The Rosenberg Self-Esteem Scale (RSES), originally developed by Rosenberg (1965) and later translated into Chinese by Ji and Yu (1993), is a widely recognized and authoritative instrument for assessing self-esteem. The scale consists of 10 items, each rated on a 4-point Likert scale ranging from 1 (strongly disagree) to 4 (strongly agree). The total score, which ranges from 10 to 40, reflects the overall level of self-esteem, with higher scores indicating higher self-esteem. The Chinese version of the scale has demonstrated good reliability, with a Cronbach’s alpha of 0.78. In this study, the scale showed a Cronbach’s alpha of 0.830, indicating good internal consistency.

### 2.6 Ethical considerations

This study received approval from the ethics committee of Xiangya Nursing School of Central South University [No: E2024114]. It adhered to the guidelines outlined in the Declaration of Helsinki. All personal and health-related information was treated with the utmost confidentiality and only used for the purpose of this research. All participants signed informed consent.

### 2.7 Data analysis

All statistical analyses were performed using R (version 4.5.0) and AMOS (version 24.0). All tests were two-tailed, and a *p*-value < 0.05 was considered statistically significant.

Descriptive statistics of the participants’ demographic characteristics were computed using R software. To assess potential common method bias, we conducted Harman’s single-factor test. Continuous variables are presented as means ± standard deviations (SD), while categorical variables are expressed as frequencies and percentages. Histograms were used to assess the normality of continuous variables. To explore potential nonlinear relationships among key variables, RCS analyses were performed. We selected the optimal number of nodes corresponding to the minimum Akaike Information Criterion (AIC) according to the AIC criterion which indicates the best model fit, and the optimal knots was 4 in our analysis (Ankerst, 2016). When drawing restricted cubic spline plots with 4 knots in RStudio, the default values for the knots are the 5th, 35th, 65th, and 95th percentiles of the predictor variable distribution. Pearson correlation analyses were used to examine the associations among social media use intensity, online group identification, online selective exposure, and self-esteem.

Mediation analyses were conducted in AMOS 24.0 to test whether online group identification and online selective exposure served as sequential mediators in the relationship between social media use intensity and self-esteem. The significance of the mediation effects was tested using the bootstrap method with 5,000 resamples, and bias-corrected 95% confidence intervals were calculated. Model fit was assessed using multiple standard goodness-of-fit indices, including the chi-square to degrees of freedom ratio (*χ*^2^/df), Tucker–Lewis index (TLI), relative fit index (RFI), incremental fit index (IFI), comparative fit index (CFI), goodness-of-fit index (GFI), normed fit index (NFI), and the root mean square error of approximation (RMSEA). Values of IFI, GFI, NFI, CFI, TLI, and RFI greater than 0.90, together with RMSEA values below 0.05 and *χ*^2^/df values between 1 and 5, were considered indicative of good model fit (Wu, 2010).

## 3 Results

### 3.1 Participant demographics

In this study, a total of 5,210 electronic questionnaires were returned, of which 4,789 were valid, resulting in an effective response rate of 91.9%. To assess common method bias, Harman’s single-factor test was conducted. The exploratory factor analysis revealed five factors with eigenvalues greater than 1, and the first factor accounted for 24.42% of the variance, which is well below the 40% threshold (Tang and Wen, 2020). In this study, a total of 5,210 electronic questionnaires were returned, of which 4,789 were valid, resulting in an effective response rate of 91.9%. The mean age of the participants was 19.8 years (standard deviation [SD] = 1.4). The sample was predominantly female (68.5%), with males accounting for 31.5%. Most participants were undergraduates, particularly freshmen (50.8%) and sophomores (33.3%), while juniors (10.7%), seniors (2.8%), and postgraduates (2.4%) constituted smaller proportions. Liberal arts majors represented the largest academic group (38.8%), followed by medicine (33.7%) and science and engineering (24.9%). Most participants were not only children (83.2%), over half reported a rural residence (59.2%), and approximately one third (34.1%) reported having left-behind experiences during childhood.

The mean age of the participants was 19.8 years (standard deviation [SD] = 1.4). The sample was predominantly female (68.5%), with males accounting for 31.5%. Most participants were undergraduates, particularly freshmen (50.8%) and sophomores (33.3%), while juniors (10.7%), seniors (2.8%), and postgraduates (2.4%) constituted smaller proportions. Liberal arts majors represented the largest academic group (38.8%), followed by medicine (33.7%) and science and engineering (24.9%). Most participants were not only children (83.2%), over half reported a rural residence (59.2%), and approximately one third (34.1%) reported having left-behind experiences during childhood.

Table 1 presents differences in social media use intensity, online group identity, online selective exposure, and self-esteem across socio-demographic variables. Significant gender differences were observed in social media use intensity and self-esteem, with females reporting higher scores than males. Significant grade differences were found in social media use intensity, online selective exposure, and self-esteem, with higher scores generally reported by students in higher grades. Academic major was significantly associated with social media use intensity, online group identity, and online selective exposure, but not with self-esteem. Only-child status and residence showed significant differences across all four variables, with only children and students from urban areas reporting higher scores. In addition, left-behind childhood experience was significantly associated only with self-esteem, with students without such experience reporting higher levels of self-esteem.

**Table 1:** Socio-demographic characteristics (*N* = 4,789).

Variables	Social media use intensity	*t*/*F*	Online Group Identity	*t*/*F*	Online Selective Exposure	*t*/*F*	Self-esteem	*t*/*F*
Gender								
Male	17.03 ± 4.67	−3.737^**^	16.20 ± 4.394	−0.843	13.14 ± 3.47	−1.069	27.43 ± 4.07	−6.890^**^
Female	17.54 ± 4.19		16.30 ± 3.81		13.24 ± 3.02		28.32 ± 4.20	
Grade								
Freshman	17.03 ± 4.27	12.293^**^	16.21 ± 3.93	1.181	13.08 ± 3.12	4.222^*^	27.77 ± 4.00	19.934^**^
Sophomore	17.73 ± 4.32		16.30 ± 4.03		13.34 ± 3.21		28.14 ± 4.18	
Junior	17.30 ± 4.62		16.21 ± 4.17		13.15 ± 3.20		28.04 ± 4.24	
Senior	18.23 ± 4.60		16.87 ± 4.24		14.00 ± 3.25		29.40 ± 5.05	
Postgraduate	19.10 ± 4.22		16.63 ± 4.14		13.57 ± 2.92		30.89 ± 4.89	
Major								
Liberal Arts	17.41 ± 4.44	6.888^**^	16.48 ± 4.07	7.433^**^	13.30 ± 3.15	4.951^*^	28.18 ± 4.28	1.239
Science and Engineering	17.69 ± 4.41		16.43 ± 4.27		13.39 ± 3.41		27.97 ± 4.34	
Medicine	17.04 ± 4.13		15.89 ± 3.67		12.97 ± 2.97		27.92 ± 3.88	
Others	18.23 ± 4.92		16.52 ± 4.26		13.19 ± 3.24		28.10 ± 4.68	
Only child								
Yes	17.89 ± 4.63	3.680^**^	16.60 ± 4.39	2.599^*^	13.43 ± 3.29	2.154^*^	28.76 ± 4.63	5.351^**^
No	17.27 ± 4.29		16.20 ± 3.92		13.16 ± 3.14		27.90 ± 4.06	
Residence								
Rural area	17.18 ± 4.15	−3.872^**^	16.12 ± 3.80	−3.037^*^	13.10 ± 3.08	−2.918^*^	27.65 ± 3.86	−7.940^**^
City	17.67 ± 4.62		16.48 ± 4.27		13.37 ± 3.28		28.61 ± 4.53	
Left-behind experience in childhood								
Yes	17.33 ± 4.24	−0.498	16.25 ± 3.77	−0.205	13.26 ± 3.03	0.753	27.75 ± 3.89	−3.484^**^
No	17.40 ± 4.41		16.28 ± 4.12		13.18 ± 3.23		28.19 ± 4.31	

^*^P < 0.05, ^**^P < 0.01.

### 3.2 Non-linear relationship between self-esteem and social media-related variables

**Figure 1:**
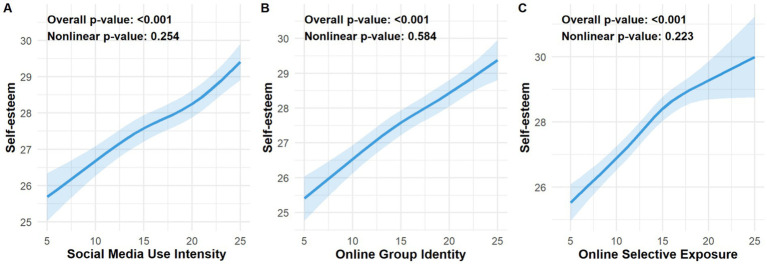
Restricted cubic spline analyses of the associations between social media-related variables and self-esteem among college students. **(A)** Association between social media use intensity and self-esteem; **(B)** Association between online group identity and self-esteem; **(C)** Association between online selective exposure and self-esteem.

To further clarify the functional form of the observed associations, multivariable-adjusted restricted cubic spline analyses were conducted, controlling for gender, age, grade, major, only-child, place of residence, and childhood left-behind experience. These analyses were used to examine potential nonlinear relationships between social media use intensity, online group identity, and online selective exposure in relation to self-esteem among college students. The findings revealed no evidence of significant nonlinear as-sociations between self-esteem and any of the three social media–related variables (Figure 1). Hypothesis 1 was not supported by the results.

### 3.3 Correlation analysis of research variables

**Figure 2:**
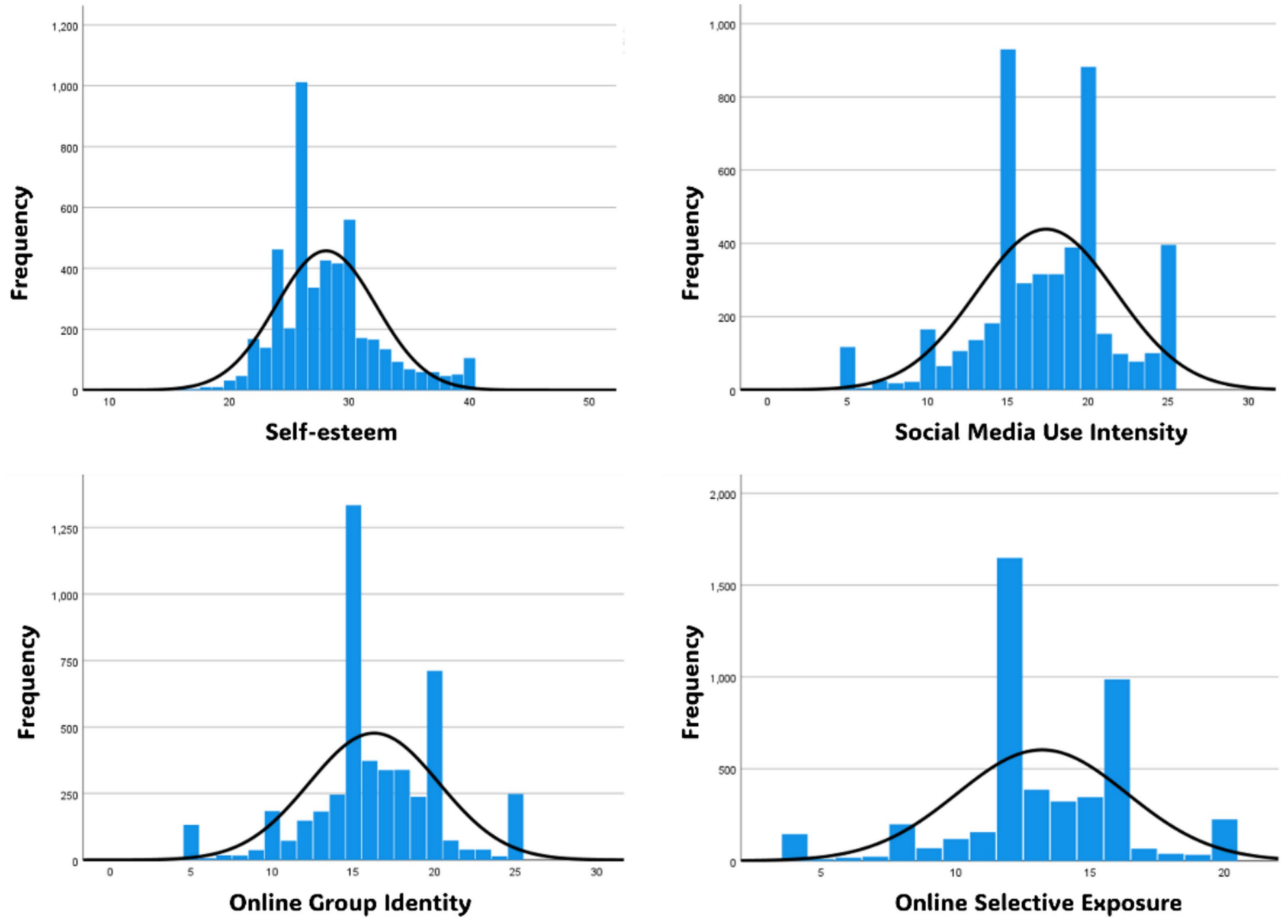
Histogram showing data distribution.

The normal curve is approximately symmetrical and bell-shaped, indicating that the data largely adhere to a normal distribution (Figure 2). As shown in Table 2, social media use intensity, online group identification, and online selective exposure were all positively and significantly correlated with one another. Social media use intensity was strongly and positively associated with online group identification (*r* = 0.682, *p* < 0.01), indicating that individuals who used the internet more frequently reported higher levels of online group identification. In addition, online selective exposure exhibited a strong positive correlation with online group identification (*r* = 0.707, *p* < 0.01), suggesting a particularly close association between these two constructs. Furthermore, self-esteem was positively and significantly correlated with all three social media–related variables. Hypothesis 2 was supported by the results.

**Table 2:** Means, standard deviations, and correlation coefficients of variables (*N* = 4,789).

	*M* ± SD	Social media use intensity	Online group identity	Online selective exposure	Self-esteem
Social media use intensity	17.38 ± 4.35	1			
Online group identity	16.27 ± 4.00	0.682^**^	1		
Online selective exposure	15.92 ± 3.76	0.616^**^	0.707^**^	1	
Self-esteem	28.04 ± 4.18	0.201^**^	0.196^**^	0.217^**^	1

^**^P < 0.01.

### 3.4 The mediating roles of online group identity and online selective exposure in the relationship between social media use intensity and self-esteem

The results indicated that the assumptions for SEM were adequately met. All observed variables exhibited acceptable multivariate normality, with skewness values below 3 and kurtosis values below 8. No multivariate outliers were identified based on Mahalanobis distance. In addition, multicollinearity was not evident in either the measurement or structural model, as all intercorrelations among observed variables were below 0.80 and variance inflation factor (VIF) values for latent constructs were less than 5.

**Figure 3:**
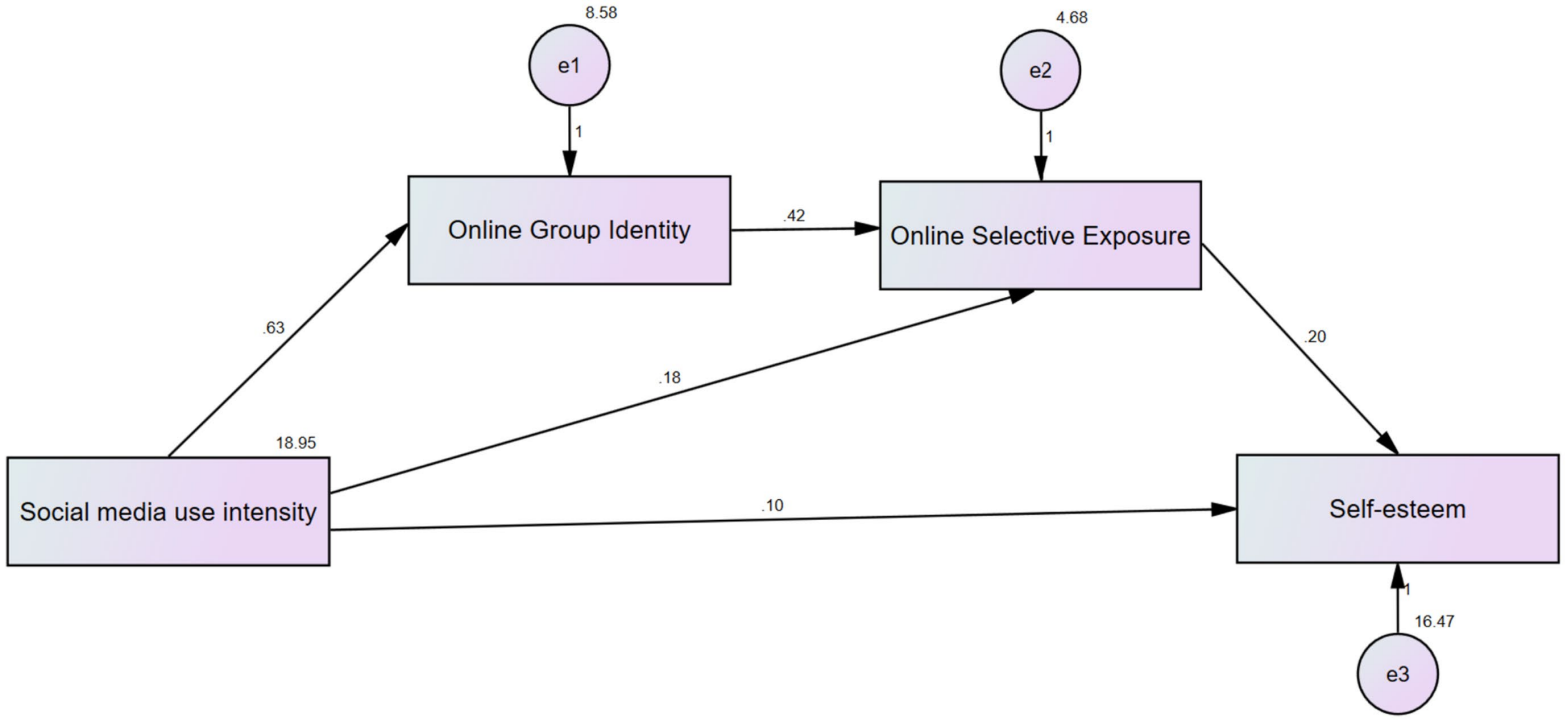
Mediating model of online group identity and online selective exposure between social media use intensity and self-esteem.

The structural equation modeling technique was employed to evaluate the proposed model. The model demonstrated a satisfactory fit, as indicated by the following fit indices: 1 < *χ*^2^/df = 3.106 < 5, TLI = 0.998 > 0.9, CFI = 1.000 > 0.9, GFI = 1.000 > 0.9, IFI = 1.000 > 0.9, RFI = 0.997 > 0.9, NFI = 1.000 > 0.9, and RMSEA = 0.021 < 0.05. All factor loading of the indicators on their respective latent constructs were statistically significant (*p* < 0.05), thereby confirming the adequacy of the model in representing the underlying constructs (Figure 3).

The direct and indirect effects were estimated using 5,000 bootstrap samples with 95% confidence intervals. As presented in Table 3, social media use intensity had a significant direct effect on self-esteem (*β* = 0.104, *p* = 0.002). Additionally, it exerted significant positive effects on both online group identity (*β* = 0.627, *p* = 0.002) and online selective exposure (*β* = 0.182, *p* = 0.002). Online group identity showed a significant positive association with online selective exposure (*β* = 0.423, *p* = 0.002), and online selective exposure further demonstrated a significant direct effect on self-esteem (*β* = 0.198, *p* = 0.002).

**Table 3:** Direct and indirect effects for the model (*N* = 4,789).

Model pathways	Estimate	Standardized effect	95% CI	*p*
Total effect	0.193	0.015	(0.162, 0.222)	0.002
Direct effect				
Social media use intensity → Self-esteem	0.104	0.020	(0.065, 0.142)	0.002
Social media use intensity → Online Group Identity	0.627	0.012	(0.603, 0.652)	0.002
Social media use intensity → Online Selective Exposure	0.182	0.013	(0.157, 0.209)	0.002
Online Group Identity → Online Selective Exposure	0.423	0.015	(0.393, 0.453)	0.002
Online Selective Exposure → Self-esteem	0.198	0.028	(0.138, 0.249)	0.002
Indirect effect				
Social media use intensity → Online Selective Exposure → Self-esteem	0.036	0.006	(0.026, 0.048)	0.001
Social media use intensity → Online Group Identity → Online Selective Exposure → Self-esteem	0.053	0.008	(0.037, 0.068)	0.001
Total Indirect effect	0.089	0.013	(0.062, 0.113)	0.002

The total indirect effect is 0.089, the total effect is 0.193, and the proportion of indirect effect = 0.089/0.193 ≈ 46.1%, indicating that approximately 46.1% of the total effect of social media use intensity on self-esteem is mediated through the indirect path, with 53.9% being the direct effect.

Furthermore, two significant indirect pathways were observed. The first indirect pathway, from social media use intensity through online selective exposure to self-esteem, was estimated at 0.036 (*p* = 0.001). The second sequential pathway, from social media use intensity through online group identity and online selective exposure to self-esteem, was estimated at 0.053 (*p* = 0.001). These results suggest that both online group identity and online selective exposure serve as partial mediators in the association between social media use intensity and self-esteem among college students. Hypothesis 3 was supported by the results.

## 4 Discussion

This study examined the associations between social media use intensity and self-esteem among college students, focusing on the mediating roles of online group identity and online selective exposure. The findings provide a nuanced understanding of how digital engagement shapes self-concept and psychological well-being during emerging adulthood.

### 4.1 Demographic influences on social media engagement and self-esteem

Univariate analyses revealed that several demographic factors including gender, grade, major, only-child status, residence, and childhood left-behind experience were significantly associated with both social media behaviors and self-esteem. Female students reported higher levels of social media use intensity and self-esteem than male students, consistent with previous research (Ma, 2022). Prior studies suggest that women are more likely to engage in interpersonal interaction and emotional expression through social media and to obtain social feedback and support from these platforms (Ellison et al., 2007; Ma, 2022). Such interactions may help satisfy social needs and contribute to higher levels of self-esteem.

Postgraduate students also exhibited higher social media use intensity and self-esteem, similar to Fu’s findings (Fu, 2024). As individuals enter postgraduate education, their social roles become increasingly professionalized, and their academic identity may serve as an important source of social recognition (Fu, 2024; Na et al., 2025). Presenting academic achievements or professional identities on social media and receiving positive feedback may therefore contribute to enhanced self-esteem. In addition, academic major was significantly associated with social media use intensity, online group identity, and online selective exposure, but not with self-esteem. This finding suggests that differences in academic environments may shape patterns of social media engagement rather than individuals’ overall self-evaluation.

Only children and students from urban areas reported higher levels across social media-related variables and self-esteem. This may be due to their relatively greater access to family and educational resources, more stable digital infrastructure, and stronger social support networks (Ma and Sheng, 2023; Zhang and Wang, 2025). Compared with non-only children and students from rural areas, they may have more opportunities to express themselves and establish social connections through social media, thereby receiving more positive feedback that reinforces self-esteem (Allen et al., 2014). These findings further suggest that family structure and urban–rural background may influence adolescents’ psychosocial adaptation through pathways related to digital engagement and access to social resources.

In contrast, students with childhood left-behind experiences reported significantly lower self-esteem. This finding underscores the long-term psychological consequences of early emotional and social deprivation, consistent with prior research showing that prolonged parental absence during childhood is associated with impaired self-concept, reduced perceived social support, and heightened vulnerability to emotional difficulties in later life (Guang et al., 2017; Wu et al., 2021). Although social media may offer alternative avenues for social connection and self-expression, such online engagement may not fully compensate for the lack of stable emotional support experienced during critical developmental periods. As a result, individuals with left-behind experiences may be less able to translate online interactions into enduring self-worth or psychological benefits.

Based on these findings, interventions to promote healthy social media use and self-esteem among college students should be tailored to students’ background characteristics. In particular, students from rural areas or with childhood left-behind experiences may require targeted support that combines structured online engagement with offline mentoring and psychological services, in order to reduce disparities in well-being and access to social resources.

### 4.2 Linear relationship between social media use and self-esteem

Contrary to the initial hypothesis, the present study did not identify a nonlinear relationship between social media use intensity and self-esteem among college students. Instead, the results revealed a stable and positive linear association between the two variables. This finding suggests that, within the range of social media use examined in this study, increases in use intensity did not reach a threshold at which the effect weakened or reversed, but were consistently associated with higher levels of self-esteem. In other words, among Chinese college students, social media use intensity demonstrates a stable positive linear relationship with self-esteem. This result is consistent with the findings reported by Zhang et al. (2023), but does not fully align with the nonlinear relationship documented by Cingel and Olsen (2018). Such discrepancies may be attributable to differences in the social contexts of the populations under investigation. Specifically, due to structured daily routines such as classroom learning, dormitory living, and participation in various campus activities, college students typically maintain relatively stable offline social interactions, which objectively constrain the amount of time available for social media use. Under these conditions, social media engagement is less likely to fully substitute for real-world social interactions, and the potential negative consequences associated with excessive use may therefore be relatively limited.

Beyond the absence of detrimental effects, social media use appears to provide college students with an important online environment through which they can access self-affirmation resources beyond offline contexts. Specifically, social media platforms offer college students increased opportunities for online self-expression, impression management, and affirming interactions with peers (Toma and Hancock, 2013; Union, International Telecommunication, 2025). These interaction processes help reinforce positive self-evaluations and satisfy individuals’ basic psychological needs for relatedness and competence. Moreover, within today’s highly algorithm-driven digital media environment, platforms continuously amplify exposure to self-relevant content through personalized recommendation systems (Lasser and Poechhacker, 2025). Such mechanisms facilitate selective exposure to self-affirming information, reduce cognitive dissonance, and further enhance the stability of individuals’ self-concepts.

Taken together, social media can be understood as a psychologically supportive social context that provides both symbolic and interpersonal self-affirmation resources for college students. This function may be particularly salient for students whose access to self-affirmation opportunities in offline contexts is relatively limited, as online interactions can partially compensate for such constraints. Therefore, social media use should be viewed not merely as a form of everyday leisure, but also as an important psychological mechanism through which individuals negotiate identity and maintain self-esteem in the digital age.

### 4.3 Mediating roles of online group identity and online selective exposure

Chain mediation analysis confirmed that online group identity and online selective exposure jointly mediated the relationship between social media use intensity and self-esteem. Online group identity represents an individual’s sense of belonging, attachment, and identification with online communities. By fostering perceived connectedness and social inclusion, such identification satisfies emotional and relational needs, reinforces positive self-concepts, and enhances perceived social validation (Barker, 2009; Trepte and Loy, 2017). In contrast, online selective exposure reflects individuals’ tendencies to attend to, interpret, and engage with information that aligns with their existing beliefs, attitudes, or self-concept. This selective engagement stabilizes self-perception by reinforcing cognitive consistency and emotional equilibrium (Knobloch-Westerwick and Hastall, 2010).

Together, these two mechanisms illustrate how social identity construction and information selection operate in a coordinated and sequential manner to link social media engagement with self-esteem. On the one hand, increased social media use facilitates individuals’ immersion in online groups, where shared norms, values, and interactions foster a sense of belonging and collective identity (Ellison et al., 2007). This group-based identification provides a socio-emotional foundation that affirms individuals’ social value and relational significance (Zhang and Zuo, 2006). On the other hand, once such identification is established, it shapes individuals’ patterns of information engagement by orienting attention toward content that is congruent with group norms and personal self-concepts (Cingel et al., 2022; Gonzales and Hancock, 2011). Through repeated exposure to self-affirming and value-consistent information, individuals experience cumulative reinforcement of positive self-evaluations (Cingel et al., 2022). Importantly, the interplay between online group identity and selective exposure reflects a coordinated psychological process, in which social validation associated with group identification and engagement with self-congruent information jointly support cognitive consistency and positive self-evaluations. Together, these processes contribute to the consolidation of self-concept and the maintenance of self-esteem over time.

The present findings extend prior research by demonstrating that self-affirmation processes are embedded within digital environments and are influenced by algorithmic content curation, which personalizes exposure to self-relevant information (Cingel et al., 2022). From a practical perspective, these results underscore the importance of fostering adaptive and reflective social media use. Educators and mental-health professionals should encourage students to engage in constructive online communities that provide affirming feedback and cultivate awareness of the psychological implications of algorithmic personalization. For students with limited offline support, intentional and positive engagement with social media can serve as a compensatory mechanism to strengthen self-esteem, resilience, and overall psychological well-being.

## 5 Limitations

This study has several limitations that should be considered when interpreting the results. First, the cross-sectional design restricts causal inference, and longitudinal or experimental studies are needed to establish temporal relationships. Second, the study also relied on self-reported data, which may introduce recall and social desirability biases, potentially affecting the accuracy of responses. Third, excluding participants with psychiatric disorders helped make the sample more uniform, but it also reduced variability in self-esteem and digital behavior. This may have weakened the ability to detect patterns that exist in a more diverse student population, limiting the generalizability of the findings. In addition, relying on self-reported psychiatric history raises the risk of underreporting or misclassification, which could bias the results. As a result, the findings may primarily reflect the psychological patterns of students without psychiatric conditions rather than the broader college population. Fourth, although online data collection allowed broad geographic coverage, it may have affected data reliability and validity due to response biases. Students with limited internet access or lower digital literacy could have been underrepresented, potentially skewing the sample toward more digitally engaged participants. Therefore, the findings may reflect the behaviors of students who are more comfortable with digital tools rather than the broader college population. Finally, while covariates were controlled in the RCS analysis, they were not included in the SEM model due to concerns about normality and multicollinearity, suggesting that future research should incorporate these factors for a more comprehensive understanding.

## 6 Conclusion

This study demonstrates a positive association between social media use intensity and self-esteem among college students, with online group identity and online selective exposure acting as significant mediators. The findings underscore the role of self-affirmation processes in digital contexts, indicating that participation in supportive online communities and selective engagement with affirming content can promote psychological well-being. Practically, educators and mental health practitioners should foster adaptive social media use and provide targeted support for students with limited offline social resources.

## Data Availability

The datasets used and analyzed during the current study are available from the corresponding author on reasonable request. Requests to access the datasets should be directed to 1062829267@qq.com.
